# Peripheral Hole Acceptor Moieties on an Organic Dye Improve Dye‐Sensitized Solar Cell Performance

**DOI:** 10.1002/advs.201500174

**Published:** 2015-09-01

**Authors:** Yan Hao, Erik Gabrielsson, Peter William Lohse, Wenxing Yang, Erik M. J. Johansson, Anders Hagfeldt, Licheng Sun, Gerrit Boschloo

**Affiliations:** ^1^ Department of Chemistry‐Ångström LaboratoryCenter of Molecular DevicesUppsala UniversityBox 523751 20UppsalaSweden; ^2^ Organic ChemistryCentre of Molecular DevicesDepartment of ChemistryChemical Science and EngineeringRoyal Institute of Technology (KTH)SE‐10044StockholmSweden; ^3^ State Key Laboratory of Fine ChemicalsDUT‐KTH Joint Research Centre on Molecular DevicesDalian University of Technology (DUT)116024DalianChina

**Keywords:** cobalt electrolytes, dye‐sensitized solar cells, interfacial charge transfer, hole transport, recombination dynamics

## Abstract

Investigation of charge transfer dynamics in dye‐sensitized solar cells is of fundamental interest and the control of these dynamics is a key factor for developing more efficient solar cell devices. One possibility for attenuating losses through recombination between injected electrons and oxidized dye molecules is to move the positive charge further away from the metal oxide surface. For this purpose, a metal‐free dye named E6 is developed, in which the chromophore core is tethered to two external triphenylamine (TPA) units. After photoinduced electron injection into TiO_2_, the remaining hole is rapidly transferred to a peripheral TPA unit. Electron–hole recombination is slowed down by 30% compared to a reference dye without peripheral TPA units. Furthermore, it is found that the added TPA moieties improve the electron blocking effect of the dye, retarding recombination of electrons from TiO_2_ to the cobalt‐based electrolyte.

## 1 Introduction

The understanding of charge transfer dynamics in dye‐sensitized solar cells (DSCs) is of fundamental interest and the control of it is essential for the elevation of devices from the laboratory to large‐scale production. The first efficient photovoltaic device based on dye‐sensitization of semiconductor metal oxides was reported more than two decades ago.^1^ Through the design of new dyes and the development of new redox mediators, the efficiency could be improved to up to 13%.^1^, ^2^, ^3^ A key requirement for an excellent device performance is highly efficient electron injection from the excited dye and regeneration of the oxidized dye by the redox mediator. Enhancing the efficiency of DSCs is critically dependent upon maintaining near unity quantum efficiencies for these charge separation processes, while at the same time reducing the energetic losses required to drive these reactions. These beneficial electron transfer processes are in competition with the decay of the excited state of the dye to the ground state and the recombination of injected electrons with oxidized dye molecules. Recombination of electrons in TiO_2_ with the redox mediator is an additional process which can limit the overall efficiency. Great advances have been made in understanding the dynamics and the impact on device performance.^4^, ^5^, ^6^, ^7^, ^8^, ^9^

In order to slow down the recombination between injected electrons and oxidized dye molecules, one possibility is to move the positive charge in the dye further away from the metal oxide surface. While this approach has been investigated using Ru‐based sensitizers together with various external donors,^10^, ^11^ the effects of coupling an external donor to metal‐free donor–pi‐linker–acceptor (D–π–A) dyes have not been explored so far. In contrast to Ru dyes, where the primary absorption stems from a MLCT transition, the primary absorption in D–π–A dyes comes from a π→π* (typically HOMO→LUMO) transition. This difference likely imposes other design criteria on assemblies of D–π–A dyes with external donors; particularly concerning the how the two are linked.

In order to reduce energetic losses in the regeneration step in DSCs, cobalt complexes have successfully been explored as one‐electron redox mediators. They have some advantages over the iodide/tri‐iodide couple, namely weak visible light absorption, less chemically aggressive and smaller voltage losses, giving the potential for more efficient solar cells.^2^, ^12^ Nevertheless, several investigations suggested that the performance of cobalt redox mediators in DSCs is limited by rapid recombination from electrons in the TiO_2_ conduction band to the cobalt(III) species and furthermore by slow dye regeneration and mass transport problems in the mesoporous TiO_2_ electrode.^13^, ^14^ Our previous research showed that a strategy of tuning the steric properties of sensitizer can overcome this recombination limitation. Furthermore, we found that additional TPA groups on the donor part of a D–π–A dye gave good blocking effect on sensitizer.^12^, ^15^

**Figure 1:**
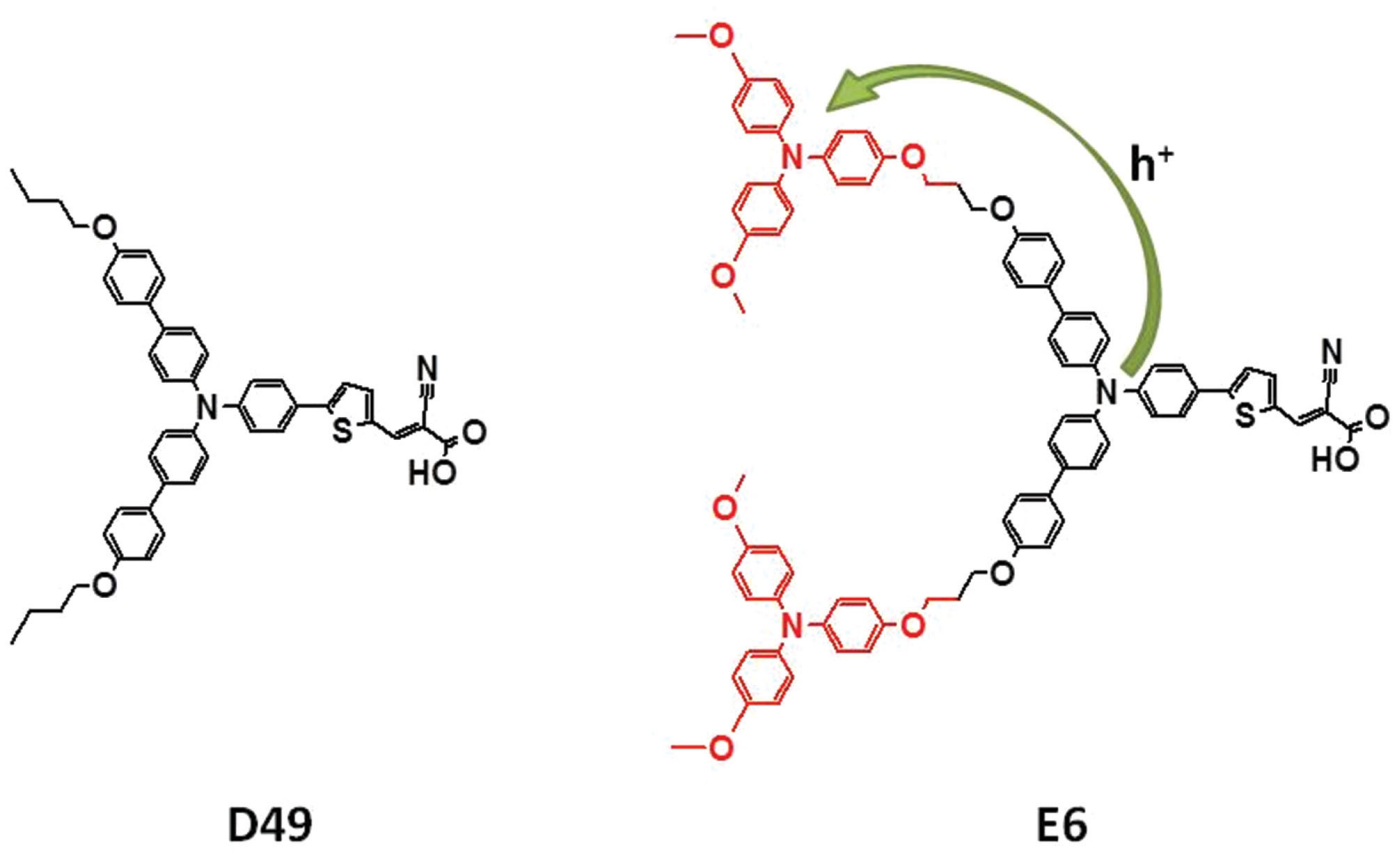
Chemical structures of D49 and E6.

Herein we design the synthesis and describe the utilization of a modified triphenylamine (TPA) dye named **E6** in which two external TPA donors via insulating aliphatic chains are introduced into donor part, in comparison to the parent dye molecule **D49**, see **Figure** **1**. After photoinduced electron injection, positive charge from the center TPA group in **E6** will move to an outer TPA unit, which will lead the charge further away from the TiO_2_ surface.

## 2 Results and Discussions

### 2.1 Synthesis of D49 and E6

**Figure 2:**
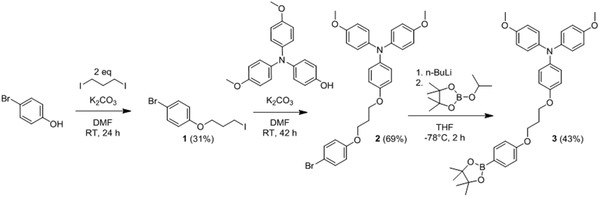
Synthesis of the external donor of the E6 dye.

**Figure 3:**
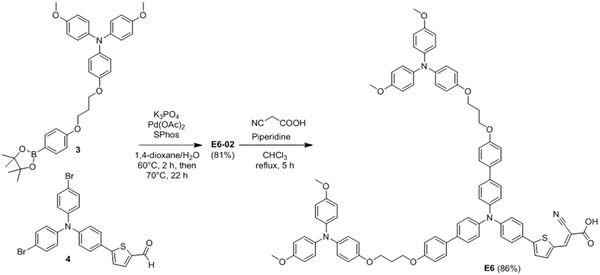
Synthesis of the E6 dye.

**Figure 4:**
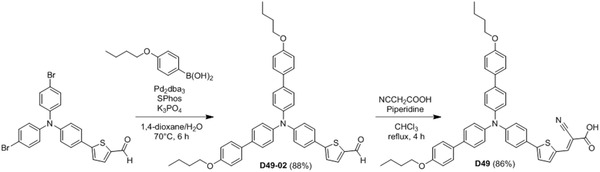
Synthesis of the D49 dye.

The synthesis of **E6** is illustrated in **Figures** **2** and **3**. A key starting material, 5‐(4‐(bis(4‐bromophenyl)amino)phenyl)thiophene‐2‐carbaldehyde (**4**), is functionalized with the external donors in good yield using the Suzuki reaction followed by a Knoevenagel condensation with cyanoacetic acid to form the desired final product (**E6**). The synthesis of **D49** is shown in **Figure** **4**, which is essentially identical to that used to synthesize D35.^16^

### 2.2 Theoretical Calculation

**Figure 5:**
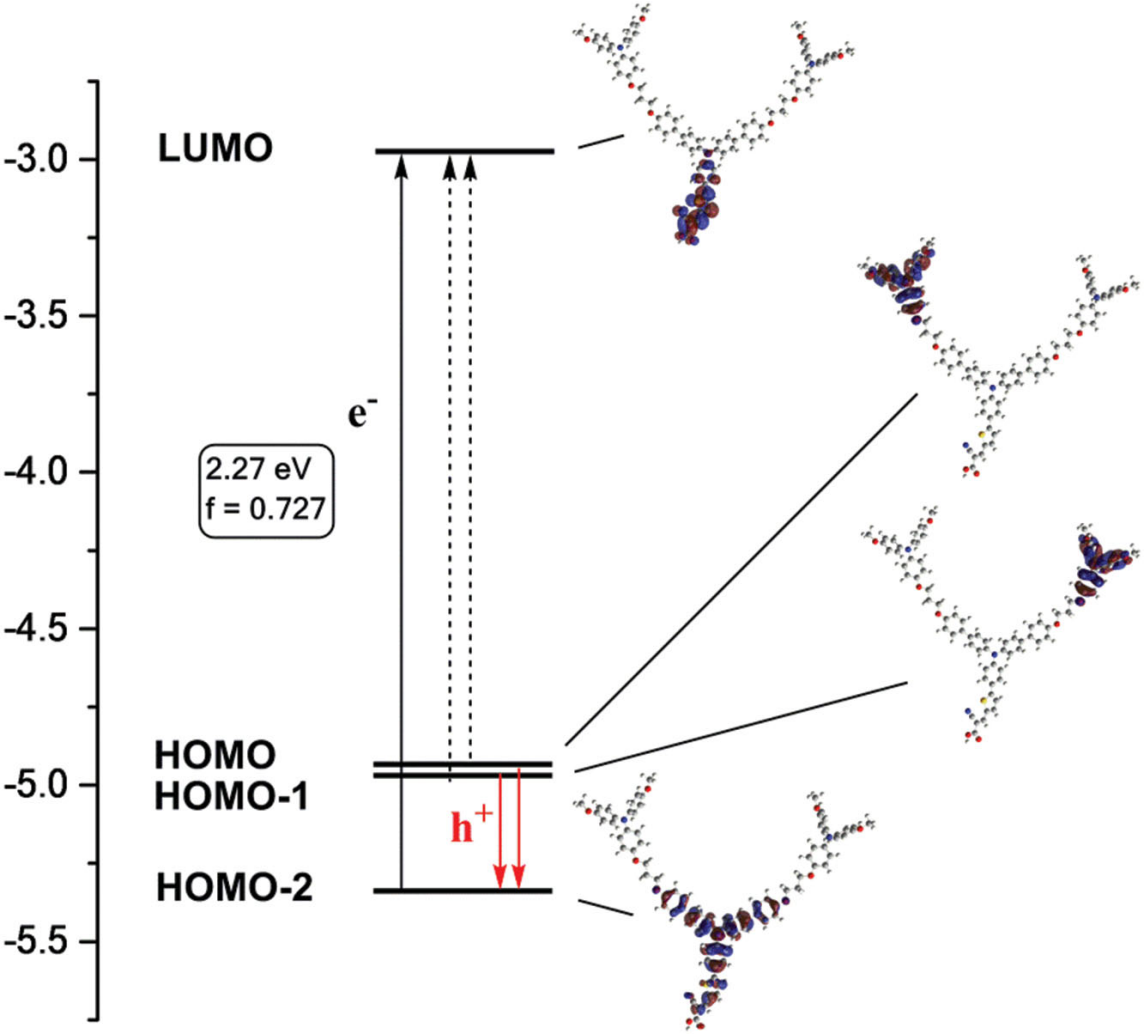
Schematic representation of the excitation energies and frontier molecular orbitals of E6 plotted at an isovalue of 0.02 from DFT calculations. The dashed lines represent forbidden transitions.

Prior to synthesis, quantum chemical calculations were performed in order to verify the dye design. The geometries were optimized using the hybrid density functional theory (DFT) functional B3LYP using the 6–31G(d) basis as implemented in Gaussian 09.^17^ A single point calculation applying a polarizable continuum model (PCM) with acetonitrile as the model solvent was then performed on the optimized structure at the B3LYP/6–311+G(d,p) level of theory. The calculation revealed two nearly degenerate energy levels at −4.970 and −4.972 eV that were assigned to the external TPA donors (**Figure** **5**). The highest occupied energy level of the dye core (HOMO‐2, −5.328 eV) lies 0.36 eV below that of the external donors', indicating a large driving force for charge transfer between the external donor and the oxidized dye. Time‐dependent DFT at the B3LYP/6–31G(d) level of theory presented two forbidden transitions (*f* < 0.0001) between the external donors (HOMO and HOMO‐1) and the dye core LUMO at 1.88 and 1.90, eV respectively. The first allowed transition (*f* = 0.727), HOMO‐2→LUMO was found at 2.27 eV.

### 2.3 Optical and Electrochemical Properties

**Figure 6:**
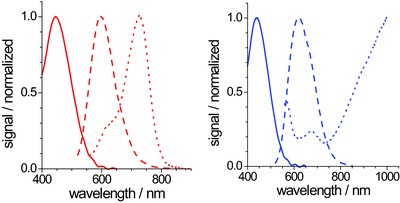
Steady‐state absorption (solid lines), fluorescence (dashed lines), and absorption of oxidized species (dotted lines) of E6 (red) and D49 (blue) adsorbed on TiO_2_, or ZrO_2_ for emission.

The steady‐state absorption, fluorescence, and oxidized dye absorption spectra of **E6** and **D49** adsorbed onto mesoporous TiO_2_ are illustrated in **Figure** **6**. The effect of extra TPA units was studied by comparison with **D49**, which does not employ these units. From a steady‐state spectroscopic point of view, both dyes behave very similarly. They both have a steady‐state absorption between 350 and 600 nm. The absorption maximum of **E6** is at 446 nm whereas it is at 438 nm for **D49**. Both dyes show strong fluorescence from the inert metal oxide surface of ZrO_2_ with a Stokes shift of 5700 cm^−1^ for **E6** and 6700 cm^−1^ for **D49**. The difference in the Stokes shift corresponds to an energy difference of 0.12 eV and implies that the excited state of **E6** is stabilized upon excitation in relation to the ground state to a higher extent compared to **D49**. The fact that fluorescence could be measured for **E6** shows that fluorescence is faster than intramolecular hole transfer to the TPA unit, which theoretically could occur. Interestingly, the absorption spectra of the oxidized dyes measured by spectroelectrochemistry are completely different as shown in Figure 6. The spectrum of the oxidized **E6** is comparable with the spectrum of pure oxidized TPA whereas the spectrum of **D49** looks similar to measurements of D35.^18^ This clearly demonstrates that the first oxidation in E6 occurs in the peripheral TPA units.

**Figure 7:**
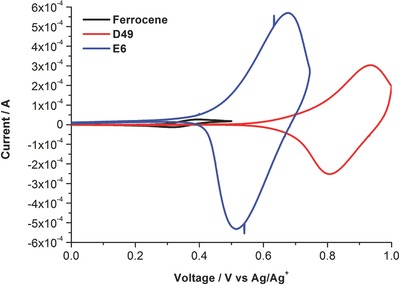
Cyclic voltammetry of D49 and E6 dyes adsorbed on mesoporous TiO_2_.

Cyclic voltammograms of **D49** and **E6** bound to mesoporous TiO_2_ are shown in **Figure** **7**. The formal reduction potentials (*E* ^0^′) were determined to be *E* ^0^′(**E6^+^/E6**) = 0.87 V and *E* ^0^′(**D49^+^/D49**) = 1.14 V versus NHE. The more negative *E* ^0^′ of **E6** is due to the introduction of the peripheral TPA electron donating (= hole accepting) groups, which are oxidized before the sensitizer core. Notably, the peak currents were about two times higher for the **E6** dye, which is in agreement with the fact that there are two TPA groups per dye molecule.

An important requirement for suppression of recombination in dye sensitized solar cells, specifically when one‐electron redox mediators are being used, is a favorable geometric position of the **E6** dye on the metal oxide surface. The additional TPA groups should point out from the surface. As the groups are connected in a flexible fashion, in the worst case scenario they could bend down close to the surface. In that case recombination might be facilitated. Photoelectron spectroscopy measurements (results shown in the Supporting Information, Figure S1) suggest that under optimized sensitization conditions using DCM as solvent, the dye orientation of **E6** on the TiO_2_ surface is favorable, with the peripheral TPA group pointing away from the surface.

### 2.4 Photovoltaic Performance of Solar Cells

**Figure 8:**
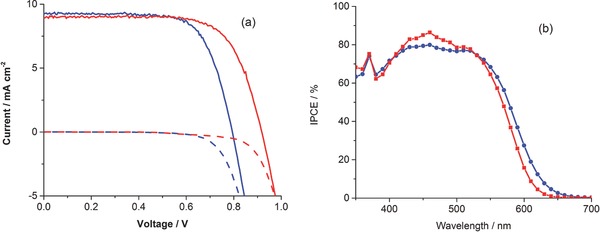
a) Current density versus applied potential curves of D46 (blue) and E6 (red) based dye‐sensitized solar cells under 1000 W m^−2^ AM 1.5 G illumination (drawn lines) and in darkness (dashed lines) and b) IPCE spectra of D46 (blue) and E6 (red) based DSCs. A cobalt‐based electrolyte is used.

In the following, we compare and discuss solar cell characterization employing both dyes. The solar cells were prepared as described before,^15^ using a paste from Dyesol with 30 nm particle size. The thickness of the TiO_2_ layer was 9 μm and the active area 5 × 5 mm^2^. A cobalt‐based electrolyte was introduced containing 0.1 M LiClO_4_, 0.2 M TBP, 0.22 M Co(bpy)_3_(PF_6_)_2_, and 0.05 M Co(bpy)_3_(PF_6_)_3_ in acetonitrile. Performance data are given in **Table** **1** and the IV curves as well as the IPCE spectra are shown in **Figure** **8**. The best overall efficiency of **E6** was 6.0% whereas it was 5.1% in the case of **D49**. From IV data it is obvious that this difference stems from a lower *V*_OC_ of **D49**. The *V*_OC_ of DSCs is given by the difference between the quasi‐Fermi level of electrons in the TiO_2_ film and the redox potential of the electrolyte. Since the redox potential of the electrolyte is the same for both types of solar cells employing D49 and E6, respectively, the lower *V*_OC_ of D49 has to be explained by a lower quasi‐Fermi level.

**Table 1:** Current–voltage characteristics of DSCs sensitized with D49 and E6 using cobalt trisbipyridine‐based electrolyte

Dye	*V*_OC_ [V]	*J*_SC_ [mA cm^−2^]	FF	*η* [%]
**D49**	0.79	9.25	0.71	5.1
**E6**	0.92	9.02	0.72	6.0

**Figure 9:**
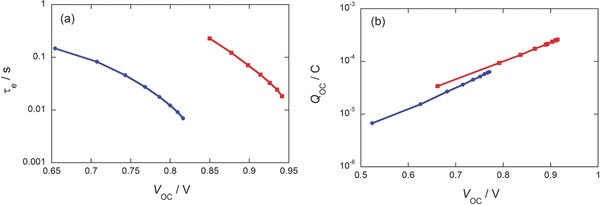
a) Electron lifetime of DSCs based on D49 (blue) and E6 (red) as a function of open‐circuit potential, recorded at different light intensities. b) Extracted charge as function of open‐circuit potential.

We performed photovoltage response time and charge extraction measurements in dependence of the light intensity in order to determine electron lifetime and possible band edge shift. As shown in **Figure** **9**, the electron lifetime of **D49**‐sensitized solar cells is much smaller at a given potential than that of **E6**, corresponding to a higher recombination rate of electrons in the TiO_2_ with Co(III) species in the electrolyte.^19^ This is also supported by the higher dark current densities found for **D49** compared to **E6** at a given applied potential, see Figure 8a. The peripheral TPA groups seem to decrease the recombination rate due to steric hindrance, keeping the Co(III) species further away from the TiO_2_ surface. Such a blocking effect of TPA groups was also recently observed for a series of phenoxazine‐based dyes.^15^

Charge extraction measurements (Figure 9b) were performed to assess the effect of the sensitizer on the energy levels of the mesoporous TiO_2_ electrode. Since no significant difference is found, it may be assumed that the conduction band edge position of TiO_2_ is not changed when sensitized with either **D49** or **E6** dye.

### 2.5 Dye Recombination and Regeneration Dynamics

**Figure 10:**
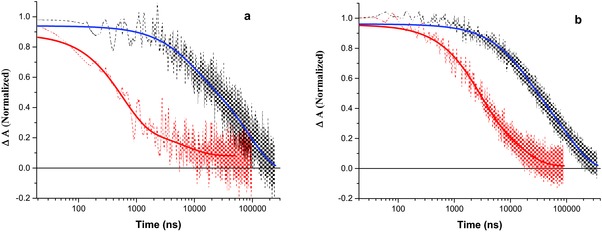
Transient absorption kinetics of TiO_2_ sensitized with a) **D49** and b) **E6** under the condition of inert electrolyte (black line) and cobalt bipyridine electrolyte (red line).

In ultrafast spectroscopy studies published previously, it was shown that after photoinduced electron injection from **E6** into TiO_2_, the hole from the core chromophore is transferred to one of the peripheral TPA units with a time constant of 75 ps.^20^ Here, we investigated the transient absorption kinetics of **D49** and **E6** sensitized TiO_2_ electrodes in air or in contact with redox couple on the nano to millisecond timescale, see **Figure** **10** and **Table** **2**. Under the conditions used, the transfer of electrons from the TiO_2_ conduction band to oxidized **E6** molecules occurred with a half time (*t*_1/2_) of 31.6 μs. In comparison, for **D49**‐sensitized TiO_2_ electrodes this process occurred significantly faster, with a half time of 23.6 μs. The decrease in recombination rate constant is mainly attributed to the increased distance between hole in the dye molecule and the electron in TiO_2_. Furthermore, the decrease in driving force for recombination may play a role. Slowing down the electron‐oxidized dye recombination kinetics in DSC will be beneficial for its performance, as the concentration of electrons in the mesoporous TiO_2_ is high under operational conditions, and electron‐oxidized dye recombination will give significant losses.

**Table 2:** Measured halftimes and calculated regeneration efficiencies for D49 and E6 sensitized DSCs employing cobalt bipyridine redox couple

Dyes	*t*_1/2_ [μs]		*Φ*_reg_
	Inert	Cobalt	
**D49**	23.6	0.5	0.98
**E6**	31.6	2.8	0.91

The regeneration half time for E6 was determined to be 2.8 μs in the presence of cobalt‐based electrolyte. This was significantly longer than that of D49, 0.5 μs under identical experimental conditions (see Table 2). The slower regeneration kinetics of **E6** is in principle unfavorable for the DSCs. The reason could be the lower driving force for the regeneration process for E6, being 0.31 V, compared to 0.58 V for D49 (the driving force is given by the difference in formal reduction potentials for the dye (E^0^′(*D* ^+^/*D*)) and the redox couple (E^0^′(Co(bpy)_3_^2+/3+^ = 0.56 V versus NHE). The overall calculated regeneration efficiency^18^ was calculated to be quite similar for both dyes, over 90%. This is consistent with the comparable maximum IPCE values found for both dyes.

## 3 Conclusion

In conclusion, E6, an organic dye with peripheral hole acceptor moieties, has been successfully used in DSCs. The hole transfer from the chromophore core to external TPA groups leads to lower electron recombination to the oxidized form of the dye. Additionally, recombination of electrons from TiO_2_ to the cobalt‐based electrolyte was suppressed; an effect that we assign to its bulky structure. The presented molecular sensitizer design, where a chromophore is linked to peripheral hole acceptors, can lead to more favorable electron transfer dynamics and improved DSC performance.

## 4 Experimental Section

*General Consideration*: All solvents and chemicals with the exception of the cobalt complexes were purchased from Sigma Aldrich and used without further purification. The cobalt complexes were obtained from Dyenamo AB.

*Synthesis and Characterization*: ^1^H and ^13^C NMR spectra were recorded on a Bruker 500 MHz instrument. The residual signals, i.e., −*δ* = 7.26 ppm and 77.0 ppm from CDCl_3_, *δ* = 2.50 and 39.4 ppm from [D_6_]‐DMSO and *δ* = 2.05, 29.84, and 206.26 ppm from [D_6_]‐acetone were used as internal references for the ^1^H and ^13^C spectra, respectively. 1,4‐diazabicyclo[2.2.2]octane (DABCO) was added to the NMR‐samples recorded in CDCl_3_ to prevent oxidation of the TPA and appears around *δ* = 3.76 ppm and 45 ppm in the ^1^H and ^13^C spectra, respectively. HRMS were recorded on a Bruker microTOF (ESI‐TOF MS) mass spectrometer. Dimethylformamide (DMF) and Tetrahydrofuran (THF) were dried by passing through a solvent column composed of activated alumina. *N*‐Bromosuccinimide was purified by recrystallization from water. Chemicals were purchased from Sigma‐Aldrich. Commercially available reactants were used without further purification unless otherwise noted. 5‐(4‐(bis(4‐bromophenyl)amino)phenyl)thiophene‐2‐carbaldehyde was prepared according to the literature procedure.^16^ Flash chromatography was performed using silica gel 60 Å (35–63 μm).

1‐bromo‐4‐(3‐iodopropoxy)benzene (1). To a stirred solution of 4‐bromophenol (1.73 g, 10 mmol) in dry DMF (20 mL) under N_2_, 1,3‐diiodopropane (2.29 mL, 20 mmol), and K_2_CO_3_ (2.07 g, 15 mmol) was added. The mixture was kept under constant stirring at room temperature for 24 h. The reaction mixture was then partitioned between Et_2_O (100 mL) and H_2_O (100 mL). The ether phase was collected and washed with H_2_O (100 mL) twice, dried over anhydrous MgSO_4_, and the solvent removed by rotary evaporation. Flash chromatography over silica gel eluting with DCM/pentanes (1:10) afforded **1** (1.05 g, 31%) as a clear oil. ^1^H NMR (500 MHz, CDCl_3_) *δ* 7.38 (d, *J* = 9.0 Hz, 2H), 6.79 (d, *J* = 8.9 Hz, 2H), 4.01 (t, *J* = 5.8 Hz, 2H), 3.36 (t, *J* = 6.7 Hz, 2H), 2.26 (p, *J* = 6.2 Hz, 2H). ^13^C NMR (126 MHz, CDCl_3_) *δ* 157.95, 132.46, 116.50, 113.27, 67.69, 32.95, 2.39.

4‐(3‐(4‐bromophenoxy)propoxy)‐N,N‐bis(4‐methoxyphenyl)aniline (2). A solution of 1 (0.944 g, 2.77 mmol) and 4‐(bis(4‐methoxyphenyl)amino)phenol (642 mg, 2 mmol) in dry DMF (10 mL) was stirred together with K_2_CO_3_ (0.828 g, 6 mmol) under N_2_ for 42 h The reaction mixture was partitioned between Et_2_O (100 mL) and H_2_O (100 mL) and the ether phase was collected. The ether phase was washed with H_2_O (100 mL) twice, dried over anhydrous MgSO_4_, and the solvent removed by rotary evaporation. Flash chromatography over silica gel eluting with DCM/pentanes (1:1 followed by 2:1) afforded **2** (0.741 g, 69%) as a pale yellow oil. ^1^H NMR (500 MHz, CDCl_3_) *δ* 7.35 (d, *J* = 9.0 Hz, 2H), 6.94 (m, 6H), 6.77 (m, 8H), 4.10 (dd, *J* = 14.2, 6.1 Hz, 4H), 3.76 (s, 6H), 2.22 (m, 2H). ^13^C NMR (126 MHz, CDCl_3_) *δ* 158.12, 155.06, 154.18, 142.29, 142.08, 132.35, 124.97, 124.83, 116.43, 115.28, 114.63, 112.95, 64.83, 64.65, 55.63, 29.42.

4‐methoxy‐N‐(4‐methoxyphenyl)‐N‐(4‐(3‐(4‐(4,4,5,5‐tetramethyl‐1,3,2‐dioxaborolan‐2‐yl)phenoxy)propoxy)phenyl)aniline (3). To a stirred solution of 2 (0.695 g, 1.30 mmol) in dry THF (20 mL) under N_2_ at −78 °C, *n*‐butyllithium (2.5 M in hexanes, 0.780 mL, 1.95 mmol) was added dropwise. The reaction mixture was stirred for 30 min at −78 °C before 2‐isopropoxy‐4,4,5,5‐tetramethyl‐1,3,2‐dioxaborolane (0.530 mL, 2.60 mmol) was added dropwise. Stirring was continued at −78 °C for 2 h, after which the solution was allowed to warm to room temperature over the course of 1.5 h. The reaction was quenched by addition of NH_4_Cl (sat) solution and extracted thrice with Et_2_O. The ether phase was dried over anhydrous MgSO_4_ and the solvent removed by rotary evaporation. Flash chromatography over silica gel eluting with pentanes/DCM (1:1) followed by DCM afforded **3** (0.325 g, 43%) as a white solid. ^1^H NMR (500 MHz, CDCl_3_) *δ* 7.74 (d, *J* = 8.6 Hz, 2H), 6.95 (m, 6H), 6.90 (d, *J* = 8.6 Hz, 2H), 6.78 (m, 6H), 4.18 (t, *J* = 6.1 Hz, 2H), 4.11 (t, *J* = 6.0 Hz, 2H), 3.77 (s, 6H), 2.24 (m, 2H), 1.33 (s, 12H). ^13^C NMR (126 MHz, CDCl_3_) *δ* 161.62, 155.05, 154.30, 142.24, 142.14, 136.66, 124.96, 124.92, 115.33, 114.65, 114.00, 83.68, 68.11, 64.81, 64.41, 55.65, 29.48, 25.00.

5‐(4‐(bis(4′‐(3‐(4‐(bis(4‐methoxyphenyl)amino)phenoxy)propoxy)‐[1,1′‐biphenyl]‐4‐yl)amino)phenyl)thiophene‐2‐carbaldehyde (E6–02). A solution of 5‐(4‐(bis(4‐bromophenyl)amino)phenyl)thiophene‐2‐carbaldehyde **4** (51.3 mg, 100 μmol), **3** (146 mg, 250 μmol), K_3_PO_4_ (212 mg, 1 mmol), Pd(OAc)_2_ (2.2 mg, 10 μmol), and 2‐dicyclohexylphosphino‐2′,6′‐dimethoxybiphenyl (8.2 mg, 20 μmol) in 1,4‐dioxane (10 mL) and H_2_O (2 mL) was stirred at 60 °C under N_2_ for 2 h. The temperature was raised to 70 °C and the reaction was allowed to run for an additional 22 h. The reaction mixture was partitioned between Et_2_O and H_2_O, the ether phase was collected and washed with H_2_O once. Removal of the solvent by rotary evaporation followed by flash chromatography over silica gel eluting with DCM afforded **E6–02** (102 mg, 81%) as an orange solid. ^1^H NMR (500 MHz, Acetone) *δ* 9.91 (s, 1H), 7.93 (d, *J* = 3.9 Hz, 1H), 7.71 (d, *J* = 8.6 Hz, 2H), 7.60 (t, *J* = 8.1 Hz, 8H), 7.56 (d, *J* = 3.9 Hz, 1H), 7.21 (d, *J* = 8.5 Hz, 4H), 7.13 (d, *J* = 8.6 Hz, 2H), 7.04 (d, *J* = 8.7 Hz, 4H), 6.90 (m, 16H), 6.83 (d, *J* = 8.9 Hz, 8H), 4.24 (t, *J* = 6.1 Hz, 4H), 4.17 (t, *J* = 6.1 Hz, 4H), 3.75 (s, 12H), 2.25 (m, 4H). ^13^C NMR (126 MHz, Acetone) *δ* 183.70, 159.68, 156.33, 155.51, 154.39, 149.99, 146.71, 143.17, 142.99, 142.82, 139.44, 137.30, 133.70, 128.65, 128.52, 128.38, 127.47, 126.43, 125.83, 125.65, 124.58, 123.52, 116.31, 115.99, 115.58, 65.60, 65.48, 55.86.

(E)‐3‐(5‐(4‐(bis(4′‐(3‐(4‐(bis(4‐methoxyphenyl)amino)phenoxy)propoxy)‐[1,1′‐biphenyl]‐4‐yl)amino)phenyl)thiophen‐2‐yl)‐2‐cyanoacrylic acid (E6). A solution of **E6–02** (82.3 mg, 65.2 μmol), cyanoacetic acid (16.6 mg, 195 μmol), and piperidine (38.8 mg, 456 μmol) in CHCl_3_ (10 mL) was refluxed for 5 h under N_2_. The reaction mixture was allowed to cool before DCM (30 mL) and HCl (1 M, 30 mL) was added. The DCM phase was collected and the solvent removed. Flash chromatography over silica gel eluting with DCM followed by acetone/DCM (1:20) and MeOH/DCM (1:20) afforded **E6** (75 mg, 86%) as a dark red solid. ^1^H NMR (500 MHz, DMSO) *δ* 8.47 (s, 1H), 7.99 (d, *J* = 4.1 Hz, 1H), 7.70 (d, *J* = 8.6 Hz, 2H), 7.64 (d, *J* = 4.0 Hz, 1H), 7.59 (dd, *J* = 12.3, 8.7 Hz, 8H), 7.16 (d, *J* = 8.4 Hz, 4H), 7.07 (d, *J* = 8.6 Hz, 2H), 7.02 (d, *J* = 8.7 Hz, 4H), 6.84 (m, 24H), 4.16 (t, *J* = 6.0 Hz, 4H), 4.09 (t, *J* = 6.0 Hz, 4H), 3.70 (s, 12H), 2.16 (m, 4H). ^13^C NMR (126 MHz, DMSO) *δ* 163.76, 157.98, 154.64, 153.78, 153.22, 148.41, 146.61, 144.97, 141.74, 141.47, 141.31, 135.44, 133.57, 131.85, 127.53, 127.44, 127.37, 125.60, 125.21, 124.52, 124.28, 124.05, 122.15, 116.62, 115.35, 114.92, 114.72, 97.29, 64.35, 64.26, 55.20, 28.73. HRMS (ESI) *m/z*: [M‐H]^−^ calcd for C_84_H_71_N_4_O_10_S, 1327.4896; found, 1327.4834.

5‐(4‐(bis(4′‐butoxy‐[1,1′‐biphenyl]‐4‐yl)amino)phenyl)thiophene‐2‐carbaldehyde (D49–02). A solution of 5‐(4‐(bis(4‐bromophenyl)amino)phenyl)thiophene‐2‐carbaldehyde (770 mg, 1.50 mmol), 4‐butoxyphenylboronic acid (873 mg, 4.50 mmol), K_3_PO_4_ (3.18 g, 15.0 mmol), Pd(OAc)_2_ (33.8 mg, 0.150 mmol) and 2‐Dicyclohexylphosphino‐2′,6′‐dimethoxybiphenyl (123 mg, 0.300 mmol) in 1,4‐dioxane (50 mL) and H_2_O (10 mL) was stirred at 70 °C under N_2_ for 6 h. The solvent was then removed by rotary evaporation. The residue was partitioned between Et_2_O and H_2_O. The ether fraction was collected, washed twice with H_2_O followed by brine and dried over anhydrous MgSO_4_. Flash chromatography over silica gel eluting with DCM afforded **D49–02** (862 mg, 88%) as an orange solid. ^1^H NMR (500 MHz, CDCl_3_) *δ* 9.85 (s, 1H), 7.71 (d, *J* = 4.0 Hz, 1H), 7.55 (d, *J* = 8.7 Hz, 2H), 7.50 (t, *J* = 8.2 Hz, 8H), 7.31 (d, *J* = 3.9 Hz, 1H), 7.20 (d, *J* = 8.5 Hz, 4H), 7.14 (d, *J* = 8.7 Hz, 2H), 6.96 (d, *J* = 8.7 Hz, 4H), 4.00 (t, *J* = 6.5 Hz, 4H), 1.79 (m, 4H), 1.51 (m, 4H), 0.99 (t, *J* = 7.4 Hz, 6H). ^13^C NMR (126 MHz, CDCl_3_) *δ* 182.74, 158.75, 154.71, 149.11, 145.63, 141.46, 137.87, 136.52, 132.88, 127.86, 127.73, 127.43, 126.36, 125.44, 123.01, 122.66, 114.95, 67.91, 31.49, 19.40, 14.01.

(E)‐3‐(5‐(4‐(bis(4′‐butoxy‐[1,1′‐biphenyl]‐4‐yl)amino)phenyl)thiophen‐2‐yl)‐2‐cyanoacrylic acid (D49). A solution of **D49–02** (831 mg, 1.27 mmol), cyanoacetic acid (325 mg, 3.82 mmol) and piperidine (756 mg, 8.89 mmol) in CHCl_3_ (30 mL) was refluxed for 4 h under N_2_. The reaction mixture was allowed to cool before HCl (1 m, 50 mL) was added and the mixture was extracted with DCM (3 × 50 mL). The solvent was removed from the combined organic phases by rotary evaporation. Flash chromatography over silica gel eluting with DCM followed by MeOH/DCM (1:20) afforded **D49** (785 mg, 86%) as a dark red solid. ^1^H NMR (500 MHz, DMSO) *δ* 13.68 (br, 1H), 8.45 (s, 1H), 7.96 (d, *J* = 3.8 Hz, 1H), 7.63 (d, *J* = 8.5 Hz, 2H), 7.59 (d, *J* = 3.8 Hz, 1H), 7.50 (dd, *J* = 15.8, 8.5 Hz, 8H), 7.06 (d, *J* = 8.3 Hz, 4H), 7.00 (d, *J* = 8.5 Hz, 2H), 6.92 (d, *J* = 8.6 Hz, 4H), 3.93 (t, *J* = 6.3 Hz, 4H), 1.68 (m, 4H), 1.42 (m, 4H), 0.92 (t, *J* = 7.4 Hz, 6H). ^13^C NMR (126 MHz, DMSO) *δ* 163.79, 158.16, 153.21, 148.30, 146.52, 144.85, 141.61, 135.39, 133.57, 131.55, 127.43, 127.32, 127.24, 125.52, 125.14, 123.96, 122.05, 116.63, 114.79, 97.29, 67.15, 30.79, 18.77, 13.71. HRMS (ESI) *m/z*: [M‐H]^−^ calcd for C_46_H_41_N_2_O_4_S, 717.2766; found, 717.2793.

*Solar Cell Assembly*: TiO_2_ photoelectrodes were prepared on fluorine‐doped tin oxide (FTO) glass, which initially was cleaned in ultrasonic bath with detergent, water, acetone and ethanol for 30 min respectively. Then the FTO glass was pretreated with TiCl_4_ (40 × 10^−3^ m in water) at 70 °C for 30 min, washed with water and dried. A screen printing technique was used to prepare the nanoporous TiO_2_ films with an area of 5 × 5 mm^2^ and a thickness of 10 μm. The film consists of one transparent layer (4 μm) which was printed with colloidal TiO_2_ paste (Dyesol DSL 30 NRD‐T) and one light‐scattering layer (5 μm) prepared by another paste (PST‐400C, JGC Catalysts and Chemical Ltd). Before printing the second layer the film was dried at 125 °C for 6 min. Afterwards the electrodes were sintered in an oven (Nabertherm Controller P320) in an air atmosphere using a temperature gradient program with four levels at 180 °C (15 min), 320 °C (15 min), 390 °C (15 min), and 500 °C (30 min). Prior to the dye‐sensitization the electrodes were post treated with TiCl_4_ as mentioned above, followed by heating at 500 °C for 30 min. At a temperature of 70 °C the electrodes were immersed in a dye bath for 4 h containing either **D49** or **E6** (0.1 × 10^−3^ m, dichloromethane). Nonattached dye was removed with dichloromethane. Counter electrodes were prepared by depositing 10 μL of a H_2_PtCl_6_ solution in ethanol (5 × 10^−3^ m) to FTO glass substrates followed by heating in air at 400 °C for 30 min. Solar cells were assembled by sandwiching the photo electrode and the counter electrode using a 25 μm thick thermoplastic Surlyn frame. An electrolyte solution was then injected through a hole predrilled in the counter electrode by vacuum back filling and the cell was sealed with thermoplastic Surlyn cover and a microscope glass coverslip. The electrolyte consists of Co(bpy)_3_(PF_6_)_2_ (0.22 m), Co(bpy)_3_(PF_6_)_3_ (0.05 m), LiClO_4_ (0.1 m) and 4‐tert butylpyridine (TBP, 0.2 m) in acetonitrile.

*Solar Cells Characterization*: Current–voltage (*I*–*V*) characteristics were determined by using a combination of a source measurement unit (Keithley 2400) and a solar simulator (Newport, model 91160). The solar simulator was giving light with AM 1.5 G spectral distribution and was calibrated to an intensity of 100 mW cm^−2^ using a certified reference solar cell (Fraunhofer ISE). On top of the DSC a black mask with an aperture of 5 × 5 mm^2^ was applied.

Incident photon to current conversion efficiency (IPCE) spectra were measured with a computer‐controlled setup comprising a xenon light source (Spectral Products ASB‐XE‐175), a monochromator (Spectral Products CM110), and a Keithley multimeter (model 2700). The IPCE spectra were calibrated using a certified reference solar cell (Fraunhofer ISE).

Electron lifetime as a function of extracted charge at different bias light intensities was investigated in a “toolbox setup” as described previously.^21^ A white LED (Luxeon Star 1W) was used as a light source. Transient photovoltage response of the DSCs was recorded using a 16‐bit resolution digital acquisition board (National Instruments) in combination with a current amplifier (Stanford Research Systems RS570) and a custom electromagnetic switching system. The transient photovoltage was recorded by overlapping the bias light with a small square wave modulation and the response was subsequently fitted to a first‐order exponential function. For the charge extraction measurements the DSCs were illuminated for 5 s at the same bias light intensities as for the electron lifetime measurements. After 5 s the LED is turned off, the external circuit is short‐circuited and the current density is read and integrated over time.

*Electrochemical Measurements*: Oxidation potentials of **D49** and **E6** attached to TiO_2_ were determined by cyclic voltammetry on a CH Instruments 660 potentiostat using a three‐electrode setup. Therefore dye‐sensitized TiO_2_ films on FTO glass were used as the working electrode, a graphite rod (3 mm) as counter electrode, and a reference electrode consisting of Ag/AgNO_3_ (10 × 10^−3^ m AgNO_3_, 0.1 m TBAPF_6_ in acetonitrile). The reference electrode was calibrated versus ferrocene in acetonitrile containing 0.1 m TBAPF_6_ (*E* ^0^′(Fc^+^/Fc) = 0.630 V vs NHE). The measurements were carried out on dye sensitized TiO_2_ films by using 0.1 m lithium perchlorate (LiClO_4_) in acetonitrile as supporting electrolyte. The scan rate was 10 mV s^−1^.

*Steady‐State Absorption and Emission*: TiO_2_ films for steady‐state absorption measurements were prepared by doctor blading colloidal TiO_2_ paste (Dyesol DSL 18NR‐T) diluted with 2‐propanol (50 wt% paste and 40 wt% solvent) on microscope glass slides and sintering at 500 °C. Absorption spectra were recorded on a Varian Cary 5000 spectrometer with baseline correction. Fluorescence spectra were detected on ZrO_2_ films using Horiba Jobin‐Yvon Fluorolog‐3. Fluorescence data was internally corrected for the instrument response function. The ZrO_2_ paste was prepared as described in the literature and spread on microscope glass slides by doctor blading technique. The thickness of the metal oxide films was about 2 μm.

*Nanosecond Transient Absorption Spectroscopy*: Electron recombination and regeneration to the oxidized dye were monitored using a Nd:YAG laser (Continuum Surelight II, repetition rate: 10 Hz, pulse width: 10 ns) in combination with an Optical Parametrical Oscillator (Continuum Surelite OPO Plus). Pump pulses of 550 nm for D49 and E6 were used. The intensity of the laser output was attenuated to 0.1 mJ pulse^−1^ in combination with a 10% transmittance filter (Thorlabs). In order to avoid stray light from the laser a cutoff filter (RG 715) was used in front of the detector. For the regeneration measurements, the electrolyte consisted of 0.22 m Co(bpy)_3_(PF_6_)_2_, 0.05 m Co(bpy)_3_(PF_6_)_3_, 0.1 m LiClO_4_, and 0.2 m TBP in acetonitrile. Recombination dynamics were measured with inert electrolyte which contains 0.1 m LiClO_4_ and 0.2 m TBP in acetonitrile. Measurements were performed on screen printed TiO_2_ films of 3 μm thickness which were sensitized according to already mentioned procedure. A sealed sample for recombination and regeneration measurements was applied.

## Supporting information

As a service to our authors and readers, this journal provides supporting information supplied by the authors. Such materials are peer reviewed and may be re‐organized for online delivery, but are not copy‐edited or typeset. Technical support issues arising from supporting information (other than missing files) should be addressed to the authors.

Supplementary
