# Supplementary material for: Peripheral Hole Acceptor Moieties on an Organic Dye Improve Dye‐Sensitized Solar Cell Performance
Source: Adv Sci (Weinh). 2015 Sep 1;2(11):1500174. doi: 10.1002/advs.201500174 (PMC5049646; doi:10.1002/advs.201500174)
Supplement: Supplementary file 1 — Supplementary [file ADVS-2-0g-s001.pdf]

Copyright WILEY-VCH Verlag GmbH & Co. KGaA, 69469 Weinheim, Germany, 2015.

ADVANCED SCIENCE
Open Access

Supporting Information

for *Adv. Sci.*, DOI: 10.1002/advs. 201500174

Peripheral Hole Acceptor Moieties on an Organic Dye Improve Dye-Sensitized Solar Cell Performance

Yan Hao, Erik Gabrielsson, Peter William Lohse, Wenxing Yang, Erik M. J. Johansson, Anders Hagfeldt, Licheng Sun, and Gerrit Boschloo**

((Supporting Information can be included here using this template))

Copyright WILEY-VCH Verlag GmbH & Co. KGaA, 69469 Weinheim, Germany, 2013.

Supporting Information

Peripheral Hole Acceptor Moieties on an Organic Dye Improve Dye-Sensitized Solar Cell Performance

Yan Hao,[‖] Erik Gabrielsson,[‖] Peter William Lohse, Wenxing Yang, Erik M. J. Johansson, Anders Hagfeldt, Licheng Sun*, and Gerrit Boschloo*

Photoelectron spectroscopy (PES)

The photoelectron (PES) measurements were performed at the Swedish national laboratory MAX in Lund.

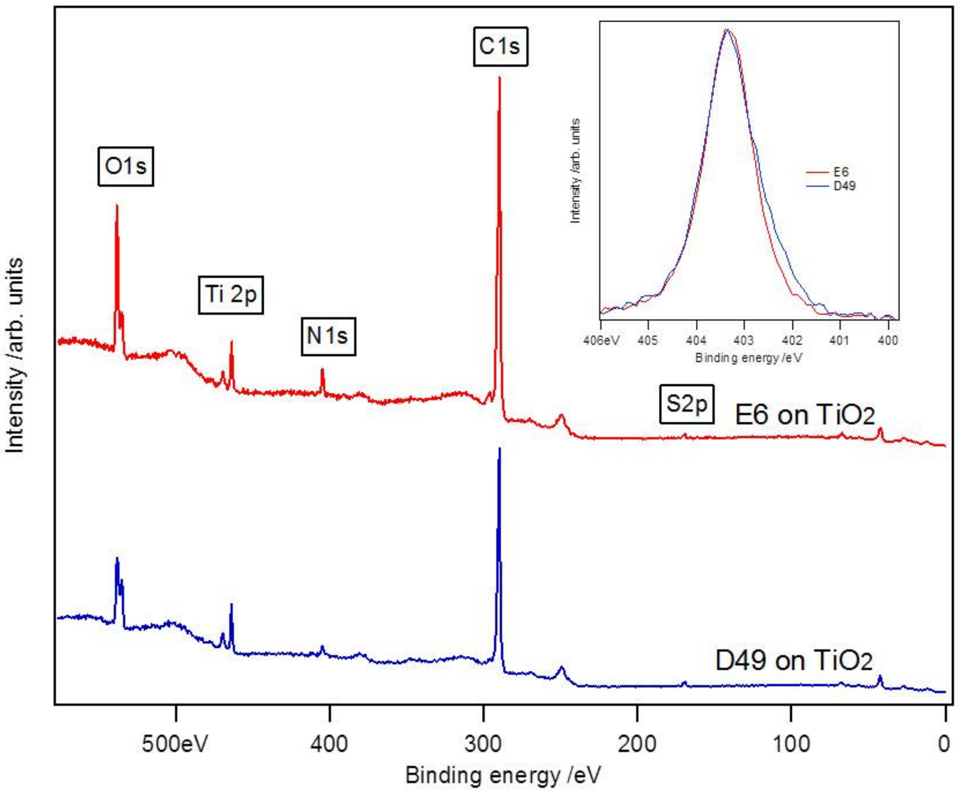

Figure S1: PES spectra of D49 (blue) and E6 (red) attached to TiO_2.

We performed photoelectron spectroscopic measurements to demonstrate that the triphenylamine moieties point out from the surface. The spectra of E6 and D49 attached to TiO_2 substrates are shown

in Figure S1. The ratio of the S2p and Ti2p peak is similar for both samples, which indicates that the coverage of the TiO_2 surface by the dye is the same for both dyes. For D49 the N1s peak shows a broadening on the low binding energy side for measurements with higher photon energies, which are less surface sensitive. This broadening comes from the CN group and is observed better with higher photon energies for a dye geometry with the CN group closest to the TiO_2. For E6, we only observe one rather narrow peak. This could mean that we need even higher photon energies to see the CN group, because of the large dye structure. Since the coverage of the surface by the dye is rather similar and we do not observe the CN group, E6 is probably standing on the surface.
